# Nudibranch color diversity shares a common physical basis in guanine photonic structure ‘pixels’

**DOI:** 10.1073/pnas.2525419123

**Published:** 2026-03-17

**Authors:** Samuel Humphrey, Xianglian He, Tobias Priemel, Vera Marie Titze, Sinuhé Perea-Puente, Vivek Subramanian, Cedric Bouchet-Marquis, Bruno Jesus, Silvia Vignolini

**Affiliations:** ^a^Department of Sustainable and Bio-inspired Materials, Max Planck Institute of Colloids and Interfaces, Potsdam 14476, Germany; ^b^Bio-inspired Photonics Group, Yusuf Hamied Department of Chemistry, University of Cambridge, Cambridge CB2 1EW, United Kingdom; ^c^Materials and Structural Analysis, Thermo Fisher Scientific, Hi6llsboro, OR 5350; ^d^Département Biologie, Institut Des Substances Et Organismes de La Mer, Nantes Université, Nantes 44322, France

**Keywords:** structural color, optics, photonics, nudibranchs, mollusk

## Abstract

Nudibranchs are well known for their bright and diverse color patterns. This coloration is typically a form of aposematism, warning predators against toxic compounds sequestered from their prey and weaponized as a form of defense. Although many of the hues in nudibranchs were thought to be of pigmentary origin, here we show, using a combination of white light and Raman microspectroscopy, that hierarchically organized micron-scale guanine multilayer structures are responsible for many of these colors. Such architectures are widespread across the dorid and aeolid groups and are responsible for a striking array of angular-independent structural colors. By using cryogenic focused ion beam (cryo-FIB) SEM tomography, we were able to access the complex 3D organization of the guanine nanoplatelets responsible for the strong blue coloration of *Chromodoris annae*. We propose that the multilayer organization of guanine platelets with varying orientations across the tissue and their micron-scale size offers a particularly effective strategy for producing diverse optical effects. The macroscopic angular independent color results from individual multilayers which we describe as “pixels”, these “pixels” reflect light at a wavelength governed by their interlayer spacing and guanine platelet thickness. The macroscopic hue can be spectrally tuned by altering the statistical distribution of pixels with each color, while the angular dependence of color can be changed through the relative orientation of the multilayer stacks and their size, allowing for a single structural motif to generate a broad palette of optical appearances.

## Significance

Nudibranchs are an extraordinarily diverse group of marine animals, renowned for their dazzling range of colors and striking patterns. While their pigmentary coloration is well understood, so far, structural coloration has been largely overlooked. In this work, we present a comparative analysis of structural coloration across nudibranch species from benthic and coral reef environments, demonstrating that guanine-based nanostructures are a widespread motif responsible for many of the brilliant colors within the dorid and aeolid groups. These nanostructures produce a unique optical mechanism, whereby matte coloration across the entire visible range can be achieved using hierarchical multilayer architectures. Microscopically, the color appears pixelated, and individual multilayers reflect a specific wavelength; these reflections are mixed at the macroscopic level, generating vibrant hues.

Nudibranchs (Heterobranchia) are often referred to as the “butterflies of the sea” (1) as they exhibit some of the most brilliantly diverse colors and patterns of all marine organisms (2–4). This coloration is thought to have evolved with shell loss, in many cases as a form of aposematism, or camouflage (4–6). They are known to be prolific kleptochemists, stealing toxins, stings, and spikes from their prey organisms. Notable examples include the “blue dragon” sea slug *Glaucus atlanticus,* which steals stinging cells (nematocysts) from the Portuguese Man o’ War Jellyfish, transferring them to its own tissue to use as a defense mechanism (7), and the “Spanish dancer” nudibranch *Hexabranchus sanguineus,* which steals chemical defenses from its sponge prey (8). A strong correlation has been shown to exist between conspicuousness and toxicity in nudibranchs (5, 9), and 50% of opisthobranchs are believed to show aposematic coloring (9).

While the extraordinary variety of their color palette is well documented, and several pigments have been proven responsible for such hues (10, 11), coloration coming from nanostructures, so-called structural color, has been largely overlooked in these species. So far, structural color has only been reported in one species of aeolid nudibranch, *Flabellina iodenea* (6). In this species, guanine crystal stacks were observed in the tips of cerata and rhinophores and are suggested to be responsible for enhancing the vividness of the pigmentary color pattern through “silvery” reflectance, as well as increasing visibility of the orange cerata at depths where penetration of light at the red end of the spectrum is low. When observed in situ through TEM, envelopes near the epithelial basal lamina contained stacks of guanine platelets free within cells and membrane-bound.

Interestingly, guanine-based structural color is found in a wide range of organisms from vertebrates, such as panther chameleons (12) and fish (13–16), to invertebrates such as spiders (15) and mollusks [e.g., the image-forming mirrors in the eyes of scallops (17)]. The crystal structure of β-guanine (the form typically found in nature) can be seen in *SI Appendix*, Fig. S1 (18, 19). Although different guanine nanostructures are responsible for structural color in a range of animals (13), vibrant matte coloration, as we report here, is uncommon. Large area ordered multilayers typically result in iridescent coloration (20–23), whereas disordered ones generate a silvery appearance (6, 15, 24). A rare example of a matte structural color produced from guanine was reported by Zhang et al. (25) in hatchling lizards. However, matte structural coloration obtained with different biomaterials has been frequently reported among birds (26–28) and beetles (29, 30) due to coherent scattering events. Similarly, both in beetles and butterflies, angle-dependent photonic crystals with polycrystalline domains can appear less macroscopically iridescent, because of optical mixing from differently oriented domains (31–35) and additional suppression of iridescence can be achieved by adding a diffusive layer to the photonic structure (36, 37). A more comparable optical response to the one we describe here is the bright blue angle-independent color in the blue-ringed octopus, where an angularly distributed array of protein multilayers is observed (38). In this example, the brightness achieved by a multilayer reflector is maintained, while its iridescence is quenched by the angular distribution of the multilayers. Protein multilayers are reported in other cephalopod mollusks but are typically iridescent (39–41). Finally, it is interesting to notice that while guanine is a common material used underwater to produce structural colors due to its high refractive index, for other gastropod mollusks, structural colors have been reported to be mineral based (42).

Here, we have studied a range of dorid and aeolid nudibranchs, demonstrating that guanine photonic structures are a common thread in producing structural coloration. These species were chosen based on their optical appearance and diversity within the two selected groups. Dorid and aeolid nudibranchs exhibit a wide range of colors and patterns, but with some key similarities (*SI Appendix*, Fig. S2). The coloration in these groups is often pixelated (composed of discrete granules) when viewed at high magnification, is described in the literature as “vivid,” “silvery,” and “iridescent,” and encompasses colors that are challenging to obtain with pigmentation (white, violet, blue) (2, 43, 44). Additionally, pigmentary analysis has thus far only been able to account for specific hues in species within patterns of multiple colors, suggesting that pigmentary color may not be the only mechanism (10, 11). Other groups, such as Dendronotoidea and Arminoidea, have not been selected as they do not display such features and are typically dull in color. The range of colors with similar optical properties, but varied patterns and hues across the dorid and aeolid groups raises the question of whether the same color generation mechanism is utilized in both cases.

To understand the role of guanine crystals in the optical appearance of nudibranchs, we used white-light microspectroscopy, while to determine the composition of the photonic structures, we used Raman spectroscopy on histological sections. Finally, we revealed the full 3D hierarchical nanoarchitecture responsible for *Chromodoris annae*’s structural coloration, using cryogenic focused ion beam scanning electron microscopy (cryo-FIB SEM). We foresee that this imaging methodology can be useful for visualizing the nanoarchitecture of guanine in a wide range of living organisms due to guanine’s excellent contrast in the secondary electron mode.

Astonishingly, we found that a single structural motif (the micron-scale guanine multilayers which act as “pixels”) can account for the optical appearance of a group of animals known to be one of the most chromatically diverse on earth. The key to this diversity stems from the optical mixing of an array of pixels of varying ultrastructure and orientation, a mechanism which is unreported in guanine systems, and more generally for fine spectral tuning of structural coloration. Such nanostructures can provide a wide range of appearances, both in terms of hue and angular dependence by simply tuning the nano and microscale arrangement of the guanine platelets. The periodicity and the guanine nanocrystal dimensions in the multilayer stack influence the color of individual pixels and their size, the relative orientation between multilayer stacks determines the level of color purity: long-range order of pixels of the same color gives rise to iridescent coloration, while a more disordered one gives rise to a matte appearance. Finally, using multilayers of different coloration at the microscale results in color mixing in the macroscopic appearance; therefore, fine color tuning can be achieved by altering the statistical distribution of the colors reflected from the individual pixels.

## Results and Discussion

### Structural Color Variation in Nudibranchs.

**Fig. 1.**
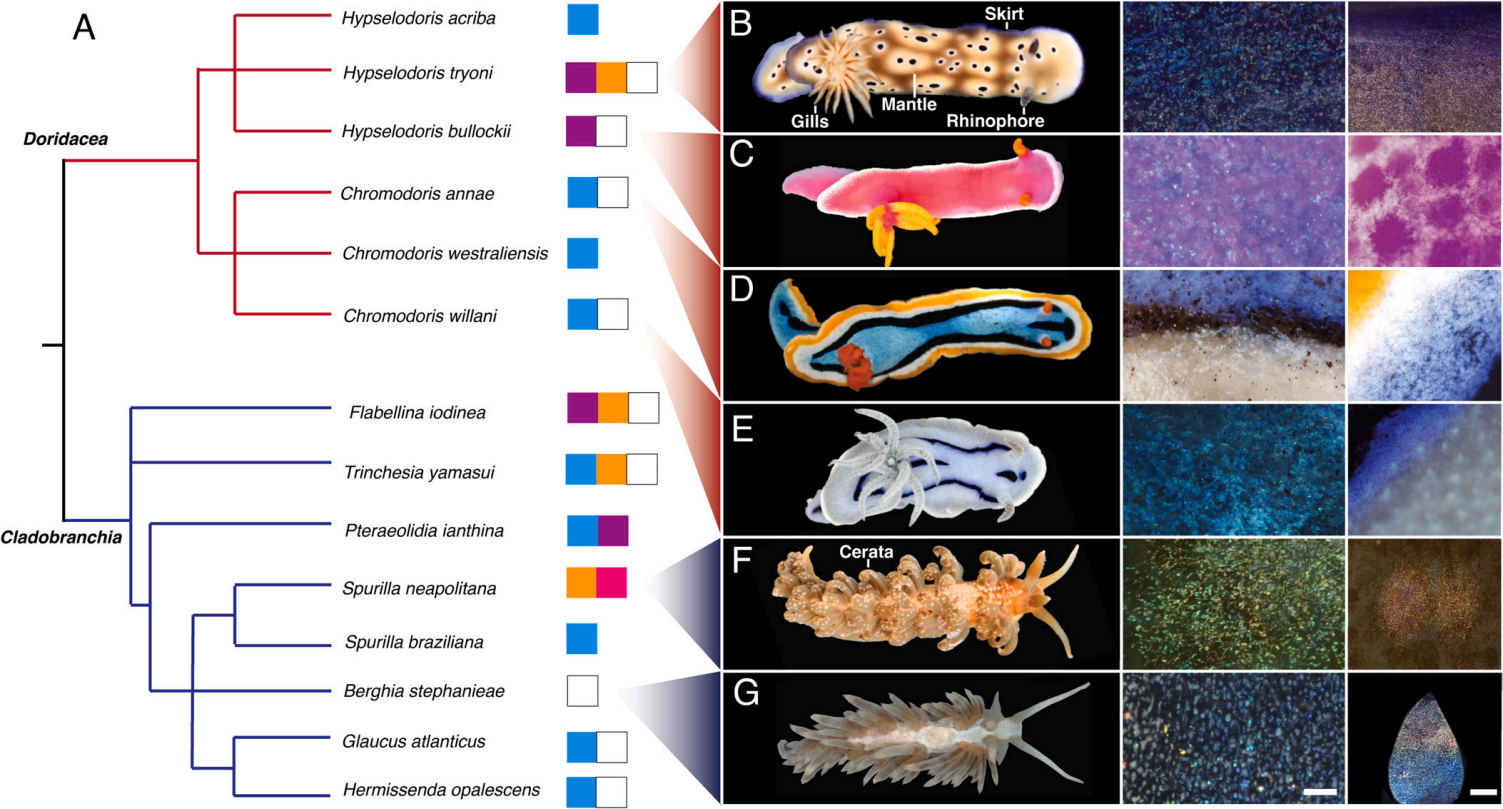
(*A*) Phylogenetic tree containing 14 nudibranch species from the clades Doridacea and Cladobranchia. The species studied here are visible in the photographs on the *Right* [from *Top* to *Bottom* (*B–G*) *H. tryoni*, *H. bullockii*, *C. annae*, *C. willani*, *S. neapolitana*, *B. stephanieae*]. Other species have either been reported to utilize guanine crystals or were selected based on their visual appearance (iridescent, brilliant, pixelated coloration on the skin) in order to show the diversity of genera believed to utilize guanine for structural color within the two highlighted clades. Images of these species can be found in *SI Appendix*, Fig. S2. The phylogenetic tree was constructed using taxonomic data from the NCBI taxonomy database. Colored squares show the range of macroscopic colors believed to be the result of guanine crystal-based structural color for each species (i.e., pigmentary colors are not included). (*B*–*G*) Digital microscope images showing structurally colored granules in *H. tryoni,* skirt (*B*), *H. bullockii* mantle (*C*), *C. annae* skirt (*D*), *C. willani* mantle (*E*), *S. neapolitana* ceras (*F*), *B. stephanieae* ceras (*G*). (Scale bar, 50 µm, *Middle* column; 200 µm, *Right* column.)

We selected 14 nudibranch species (6 dorids, 8 aeolids) and constructed a phylogenetic tree based on NCBI (National Center for Biotechnology Information) taxonomic data (Fig. 1*A*) to illustrate the distribution of color traits across the two groups (45). From this broader set, we focused on six representative species—*Hypselodoris tryoni*, *Hypselodoris bullockii*, *Chromodoris annae*, *Chromodoris willani*, *Spurilla neapolitana*, *Berghia stephanieae*—chosen for their color diversity and their phylogenetic range within the dorid and aeolid clades. As visible in Fig. 1, pixelated, iridescent-colored granules are a common thread in these species, and so, despite their varied macroscopic appearance, we expected that the mechanism of color production may be the same. Additionally, all the above dorid nudibranchs sequester secondary metabolites as chemical defenses (46–48), while the aeolid nudibranchs utilize stolen cnidocytes from anemones (49–51), so structural color may have an aposematic function in all of the species studied here.

In the four dorid nudibranchs shown here, the skirt coloration is distinct from the mantle. In all cases, there are regions of white coloration and regions of violet or blue coloration. For *H. tryoni,* the outer skirt is colored in a violet hue, while the mantle is white/beige colored with mauve spots, *H. bullockii* has a white skirt with pink/violet coloration on the mantle and at the base of the rhinophores and yellow gills/rhinophores, *C. annae* has five distinct regions of coloration with stripes of yellow, white, black, and blue on the mantle and orange gills/rhinophores, *C. willani* has a white skirt and black/blue stripes on the mantle. The two aeolid nudibranchs shown here have coloration concentrated on their cerata, in the case of *S. neapolitana* in the form of orange spots, while for *B. stephanieae,* the entire cerata appear white (however, at high magnification can be seen to show a gradient of coloration (Fig. 1 *G*, *Right*-hand column). *S. neapolitana* is colored brown by *Symbiodinium* microalgae sequestered from its anemone prey.

**Fig. 2.**
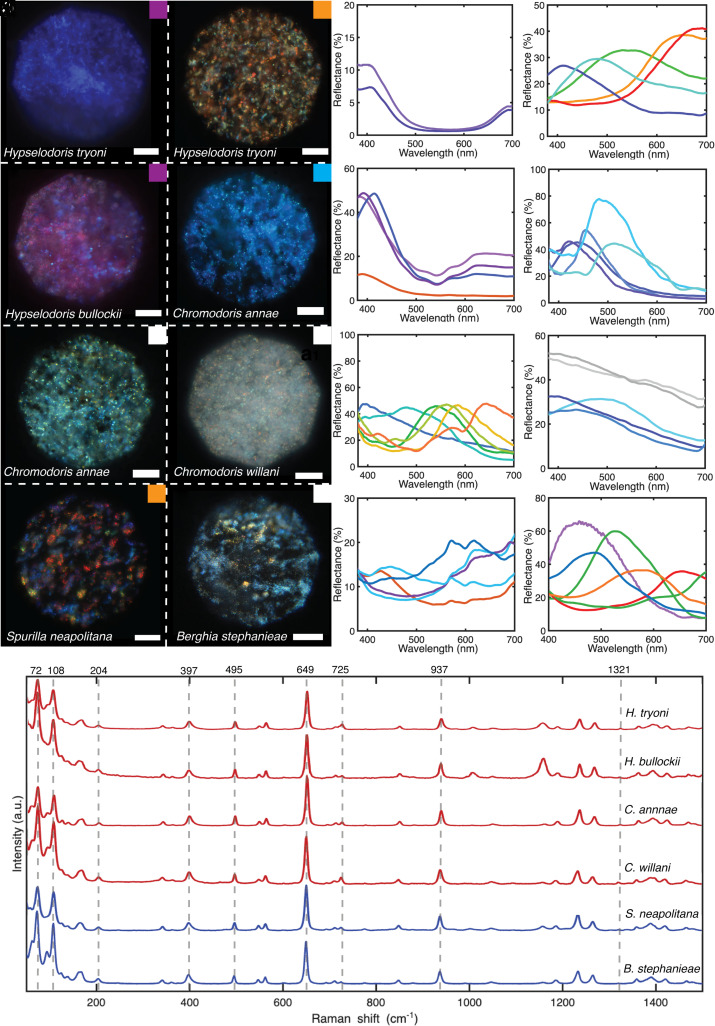
(*A*–*H*) Brightfield upright optical microscope (Zeiss axioscope, 40× water immersion objective) images of structurally colored regions on the dorsal surface of the six species shown in Fig. 1*A*. (Scale bar, 30 µm.) The color of the squares at the *Right* *Top* corner of the image corresponds to the structural coloration perceived macroscopically. (*A*) *H. tryoni* violet region, located at the perimeter of the skirt. Only violet domains of consistent color are visible here. (*B*) *H. tryoni* beige/white region. Domains of all colors are visible here, with a higher frequency of those in the orange-red wavelength range. (*C*) *H. bullockii* pink region. Domains of violet and background pink coloration are visible. (*D*) *C. annae* blue region showing granular appearance and cloudy patches. (*E*) *C. annae* white region, showing multiple domains of all colors across the visible spectrum. (*F*) *C. willani* white region. Colored domains across the visible spectrum are visible atop a cloudy white background. (*G*) *S. neapolitana* orange region (located on the cerata) showing discrete domains of color across the visible wavelength range. (*H*) *B. stephanieae* ceras. Colored domains across the visible spectrum are visible, dominated by those at the blue end of the spectrum. In all of the above images, colored domains ranging in size from 0.2 to 16 µm appear to have random orientation. (*I*–*P*) Brightfield reflectance spectra (Zeiss axioscope, 40× water immersion objective, 50 µm fibre) normalized to a silver mirror, corresponding to the colored regions shown in *A*–*H*. The overlapping reflectance spectra are for individual co-occurring granules. Peak shape, intensity, and position vary between species. Notably, white regions [*C. annae* (*E*)*, C.* willani (*F*)] appear to have spectral peaks across the visible range, suggesting a coloration mechanism similar to an LED in which colored pixels mix to give an overall white appearance. Orange/beige regions [*H. tryoni* (*B*)*, S. neapolitana* (*G*)] also show spectral peaks across the visible range, although dominated by those in the orange-red wavelength range. *C. willani* shows a broadband white spectrum when the focal plane is changed to the “cloudy” material. (*Q*) Raman spectra of iridescent regions located close to the dorsal surface in histological sections of the six nudibranch species studied here. Dashed lines indicate peak positions of literature values for anhydrous biogenic guanine and hypoxanthine.

To confirm the structural origin and characterize the optical properties of coloration in the above species, high magnification optical imaging in the Köhler illumination configuration was done on the structurally colored regions shown in Fig. 1. These regions can be clearly recognized in the microscope images of Fig. 2 *A*–*H*. In all cases, the color in the images appears pixelated; composed of discrete regions of different colors with diameters ranging from 0.2 to 16 μm. Granule diameter varies between species: *H. tryoni* —3 ± 2 μm, *H. bullockii*—7 ± 4 μm, *C. annae*—3 ± 3 μm, *C. willani*—1.0 ± 0.7 μm, *S. neapolitana—*5 ± 2 μm, *B. stephanieae*—8 ± 4 μm*. C. annae* and *C. willani* display blue coloration together with black pigmentation to increase contrast (52). Such small granules reflecting all colors will behave like pixels at a microscopic scale; therefore, due to color mixing, the final observed resolution will be the intensity-averaged sum of the color reflected from individual pixels. This effect is like the function of light-emitting diodes (LEDs) in an RGB display (53), or vibrant coloration achieved by the optical mixing of adjacent brush strokes in impressionist paintings (54) and has also been observed in other species, such as the fruits of *Pollia condensata* (55).

Reflectance spectra of individual granules were measured (all spectra are referenced to a silver mirror) for the different species. As visible in Fig. 2 *A*–*H*, different species and different regions within one species show different reflections. For example, the blue region in *C. annae* (Fig. 2*D*) has reflectance peaks with up to 80% intensity centered at wavelengths within 420 to 515 nm (Fig. 2*L*), while for the white region (Fig. 2*E*), the reflectance peaks span across the visible spectrum (395 to 645 nm) with a consistent reflectance of ~50%, leading to a white macroscopic appearance. *H. tryoni* again has two distinct structurally colored regions (Fig. 2 *A* and *B*), a violet one [reflectance peaks from 395 to 410 nm (Fig. 2*I*)] and a white/orange region, where we observed reflectance peaks from 410 to 690 nm (Fig. 2*J*), weighted toward those at longer wavelengths. This intensity difference will result in an orange/white appearance macroscopically, despite individual granules also reflecting shorter wavelengths. At high magnification, *H. bullockii* displays violet granules above a pink background (Fig. 2*C*), which results from a layer of pigment lying below the granules. The violet granules possess reflection spectra with intensity up to 50% in the wavelength region from 385 to 415 nm (Fig. 2*K*). *S. neapolitana* displays granules with color across the visible wavelength range (Fig. 2*G*). Spectral peaks are weighted by intensity toward those at longer wavelengths (Fig. 2*O*) to give an orange macroscopic appearance. The white region in *C. willani* is composed of two layers, which can be imaged at different focal planes (*SI Appendix*, Fig. S3). In Fig. 2*F*, we see structurally colored granules with reflectance across the visible range (corresponding to the blue lines in Fig. 2*N*) while in the focal plane lying above these granules, there is a diffuse white material (corresponding to the gray spectra in Fig. 2*N*). We hypothesize that the diffuse material acts to scatter light reflected from the colored domains, homogenizing the colored reflections, to give a white, rather than silvery appearance, analogous to diffuse reflectors observed in butterfly wing scales (36). While the coloration shown here is consistently composed of discrete, angular granules with random orientation, the visual appearance of the animals varies widely. We propose that this occurs by the variation in spectral properties of the individual granules (due to differences in ultrastructure), and by the statistical distribution of these spectra when averaged over many granules.

To confirm the presence of biogenic guanine crystals as reported in the nudibranch *F. iodenea,* we used Raman spectroscopy on histological sections of tissue from all the described nudibranchs: four dorid, two aeolid species. Raman is a convenient method to identify the material present in the section. Raman spectra were taken of areas that appeared to be highly reflective in the brightfield mode. Although the color of the six species is highly varied, the material used to produce this color is the same. The six species sampled have peaks at Raman shifts of 397, 495, 649, and 937 cm^−1^. These peaks match the literature values for anhydrous biogenic guanine (56). Additional smaller peaks at 725 and 1,321 cm^−1^ are visible, particularly in *C. willani* and *H. tryoni*, demonstrating the presence of hypoxanthine; this indicates the occurrence of guanine-hypoxanthine mixed crystals, consistent with previous reports (57). All species showed low wavenumber peaks distinct to guanine’s β polymorph (58) (72, 108, and 204 cm^−1^) while peaks corresponding to the α polymorph (62, 94, and 168 cm^−1^) are present in varying proportions in all species aside from *H. bullockii* and *S. neapolitana* (*SI Appendix*, Fig. S4). These results suggest that both polymorphs may occur in physical mixtures within the tissue, dominated by the β polymorph. As described in *F. iodenea* (6), guanine’s high refractive index in the (100) direction (~1.83) (59) allows high intensity reflections to be achieved with only a few layers. While the presence of guanine in other species of nudibranch is not unexpected, we believe that the diversity of colors that it is able to produce is remarkable, even within a single individual. Interestingly, for the two *Hypselodoris* species, guanine was associated with a pigment peak at a Raman shift of 1,155 cm^−1^ that decreased in intensity over time under laser illumination, relative to the guanine peaks (*SI Appendix*, Fig. S5), while for the two aeolid species, the presence of guanine crystals is associated with an unidentified fluorescent compound (*SI Appendix*, Fig. S6). These additional components verify that structural color in these species works in synergy with pigmentary coloration. Based on our Raman characterization, the color mechanism is the same in these two groups; however, the question still remains as to whether the evolution of structural color in these groups emerged in a common ancestor or via convergent evolution.

### Guanine Multilayer Stacks in *C. Annae*.

**Fig. 3.**
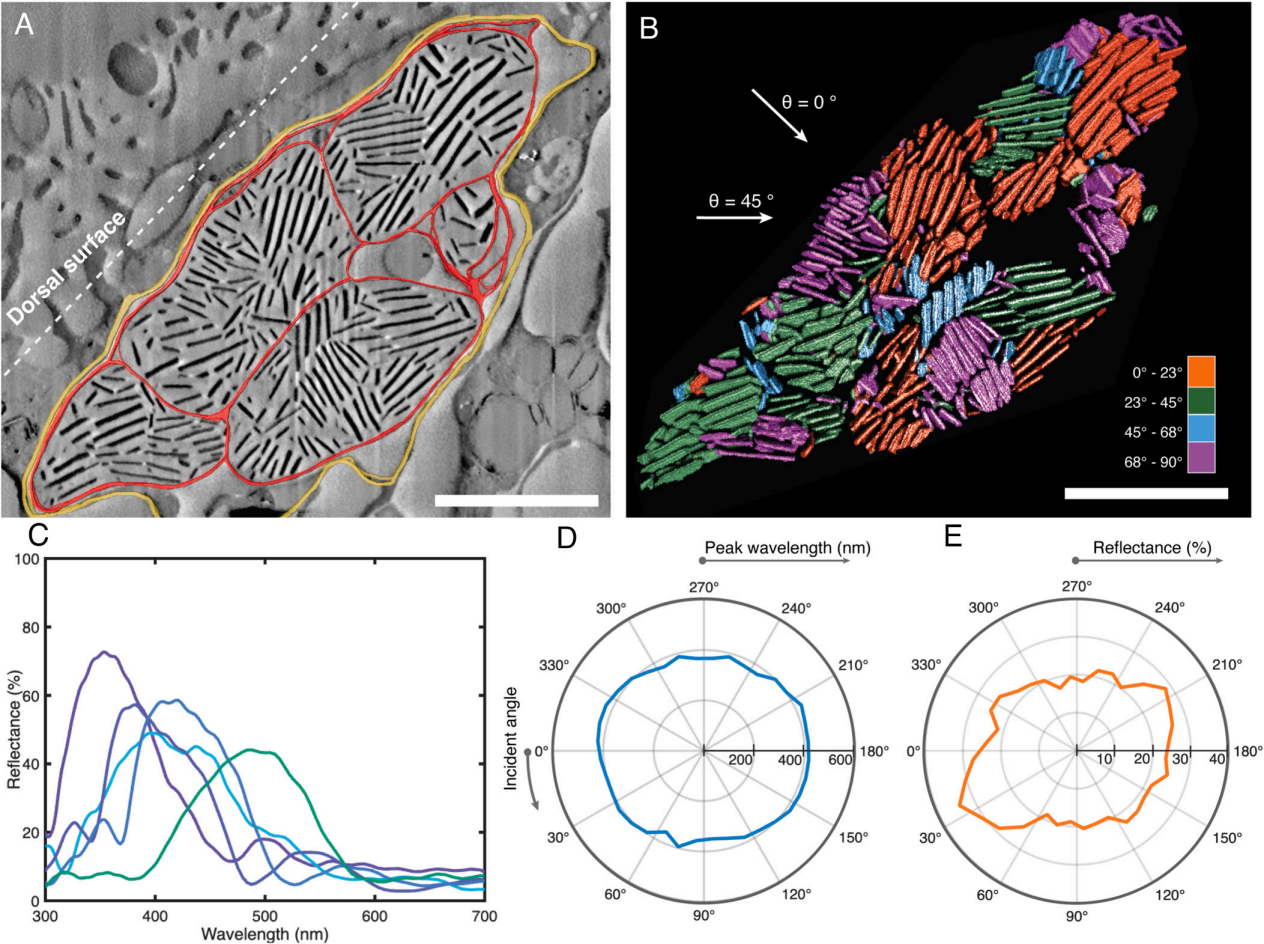
Nanoscale characterization of tissue from the blue dorsal region of *C. annae* with cryo-Plasma-FIB SDB and optical modelling. The volumes imaged here are located 2 to 20 µm from the surface of the sample. (*A*) Combined SEM image with a 3D reconstruction of outer membrane (yellow) and inner membranes (red) superimposed. Inner membranes contain multiple stacks of electron-dense crystals with apparently random orientation ranging from 1 to 16 crystals thick. (Scale bar, 3 µm.) (*B*) 3D reconstruction of the electron-dense crystals in *A*, showing the crystals are plate like in morphology. Stacks are colored by their orientation. (Scale bar, 3 µm.) (*C*) FDTD simulations of the reflectance spectra of individual guanine platelet stacks isolated from the volume in *B*, taken at normal incidence with respect to the orientation of each stack. Spectra show sharp reflectance peaks (full width at half maximum ranging from 80 to 120 nm) with peak positions ranging from 350 to 500 nm and reflectance of up to 73%. (*D*) Polar plot of reflectance spectra peak position vs. incident angle θ when illuminated over the entire reconstructed volume in *B*. The incident beam is rotated around the volume (angles are indicated in *B*), demonstrating that the spectral peak position is relatively angle independent—remaining between 350 nm and 430 nm at all angles of incidence. (*E*) Polar plot of reflectance spectra maximum amplitude vs. incident angle when illuminated over the entire reconstructed volume in *B*, corresponding to the peak positions in *D*. The amplitude varies from 17 to 34%.

In order to characterize the nanoarchitecture responsible for the structural coloration, cryo Plasma-FIB SDB (serial data block) imaging was conducted on a region of blue *C. annae* dorsal mantle tissue. Two regions of interest were imaged (Fig. 3 and *SI Appendix*, Fig. S7), finding stacks of electron-dense material in both cases, lying from 2 µm up to 20 µm from the surface of the sample. Correlating this with the Raman spectra shown in Fig. 2, we conclude that these crystals are biogenic guanine. In 2D, these electron-dense regions appear as rods (Fig. 3*A*); however, when reconstructed in 3D (Fig. 3*B* and *SI Appendix*, Fig. S7), they have a clear plate-like morphology (as expected for guanine crystals). The in-plane shape is faceted and anisotropic, forming a mixture of elongated hexagons and rhombi with lengths 0.1 to 2 µm and widths of 0.1 to 0.5 µm. These platelets form stacks of up to 16 crystals (with an average of 6 ± 3 platelets per stack), with a less electron-dense material separating them. The stacks are enclosed within inner membranes (Fig. 3*A* red), which are in close contact with each other within an outer membrane or envelope (Fig. 3*A* yellow). The diameter of the inner membrane-enclosed volume bounding the stacks varies from 1 to 3 µm, while the outer membrane encloses a volume which is ~8 µm in its long direction and 5 µm in its short direction. The envelopes containing volumes of crystalline stacks are interconnected (*SI Appendix*, Fig. S7), forming a network that extends over 10 µm in all three dimensions. The mean thickness of a platelet is 53 ± 10 nm, ranging from 39 to 68 nm, while the mean spacing of the platelets is 70 ± 24 nm, giving a total periodicity of the multilayer stacks of ~120 nm.

The optical response of a single stack of guanine platelets, such as those found through FIB SEM imaging here, can be approximated to that of a standard multilayer reflector. When the plate orientation, thickness, and spacing are uniform within the structure, reflection intensities up to 100% can be achieved with only a few layers due to the high refractive index contrast of the platelets (13). For this reason, the multilayer is an efficient structure to generate bright and brilliant colors. To verify that the structure imaged in Fig. 3*B* is responsible for the observed optical properties, we simulated the optical response of individual stacks within the volume by performing finite-difference time-domain (FDTD) simulations on the volume reconstructed from FIB SEM imaging (*Materials* *and* *Methods*). The simulated spectra contain well-defined, bright (up to 73%) reflectance peaks ranging from 350 to 500 nm (Fig. 3*C*), closely matching the experimental spectra shown in Fig. 2*L*. Our simulations confirm that this structure is responsible for the blue coloration seen in vivo.

A homogeneous multilayer structure gives rise to an angle-dependent response, where a blue shift occurs as the angle of illumination is increased. The wavelength at which the reflection is maximized in a multilayer can be calculated simply using Bragg’s law (λ = 2ndcosθ) where n = average refractive index of the layers, d = lattice constant, and θ = angle of incidence (60). Consequently, the color reflected from a perfect infinitely large individual stack shifts to lower wavelengths at higher incident angles (*SI Appendix*, Fig. S8*B*). Here, however, we observe that *C. annae* maintains its bright blue appearance (Fig. 1*D*) when viewed from any angle by varying the orientation of the multilayer stacks in space. In the reconstructed volume in Fig. 3*B*, as an example, the differently oriented multilayer stacks (shown in different colors) are broadly and evenly distributed between 0° and 90° relative to the animal’s surface, with an average angle of 50° ± 30° measured across 25 stacks (see *SI Appendix*, Fig. S9 for the full platelet angle distribution). The misorientation of the crystal stacks means that, whatever the viewing angle, there will be a multilayer stack which is oriented perpendicular to the incident light, and constructive interference will thus achieve blue reflectance. To confirm this effect, we simulated the optical response of the entire volume in Fig. 3*B*, while varying the incident angle θ, finding that at all values of θ, the peak wavelength of reflected light lies between 350 nm and 430 nm (Fig. 3*D*). The reflected intensity in this configuration varies from 17 to 34% (Fig. 3*E*). Variation in stack orientation to homogenize color from multilayer stacks has been reported in other systems, such as the blue ringed octopus (38). Such a hierarchical structure achieves angle-independent coloration from an intrinsically angle-dependent photonic structure.

We have also shown through 2D block-face SEM imaging that the dorsal surface of the dorid nudibranch *H. bullockii* contains similar misoriented guanine crystal stacks (2 to 10 platelets per stack), confined within inner and outer envelopes (*SI Appendix*, Fig. S10 *A* and *B*). In this case, the mean platelet thickness is 49 ± 7 nm and the mean spacing between platelets is 69 ± 20 nm giving a total pitch of 118 nm. This relatively smaller pitch shifts the constructive interference condition to shorter wavelengths, explaining why the reflectance peaks in Fig. 2*K* are blue-shifted from those of *C. annae* in Fig. 2*L*. Additionally, imaging the cerata of the aeolid nudibranch *B. stephanieae* (*SI Appendix*, Fig. S10 *C*–*F*) in this way demonstrated an array of guanine platelets with a mean platelet thickness of 150 ± 30 nm. Here, the platelets are stacked around the border of envelopes (~10 μm from the surface of the ceras, around the entire perimeter). The space between these platelets (which is far smaller than in the dorid species, relative to plate thickness) and their orientations are not well defined. This lack of structural coherence explains the large variation in coloration and textured appearance of the domains in this species (Fig. 2 *H* and *P*), resulting in an overall white appearance.

To give an idea of how small changes in the guanine platelet thickness and spacing can give rise to different optical responses across the visible range, we use a transfer matrix method (61) to compute guanine multilayer stacks with varying plate thickness and spacing. *SI Appendix*, Fig. S8*C* shows that by varying plate thickness from 50 to 90 nm and spacing from 50 to 110 nm for multilayer stacks containing six platelets, reflectance peaks from across the entire visible wavelength range can be achieved. These simulations support our hypothesis that the nudibranch species studied here generate a wide range of colors by varying the thickness and stacking pitch of guanine platelet stacks. On a larger scale, nudibranchs utilize these structurally tuned stacks to give pixels (domains) of different colors, which combine to give a range of colors based on their statistical distribution (33). In other words, ultrastructural tuning determines the available color palette on a microscopic scale, while the proportion of pixels with each ultrastructure determines the macroscopic color. Their angular distribution permits matte rather than iridescent coloration. Additionally, guanine’s refractive index allows for high reflectivity from stacks composed of just a few nanoplatelets, allowing for efficient packing of multiple stacks of different orientations. This permits a small pixel size, improving color purity at a macroscopic scale. Such a mechanism has not been reported so far for guanine structural color, which is typically iridescent (20–23), silvery (6, 15, 24), or white (62). In these cases, the domains composed of multilayers are typically larger than the pixels we report here, where the ability to control local orientation of adjacent multilayers is key to efficient color mixing.

Although the structural colors here are not dynamic as in cephalopods (39, 40), the vibrant, broad palette available promotes pattern diversity, and noniridescence may enhance the aposematic function, similar to the blue-ringed octopus (38). Access to the full three-dimensional distribution of these guanine architectures was key to understanding the physical mechanisms of the observed noniridescent coloration in nudibranchs. We believe that the prior lack of a 3D overview of the platelet distribution has meant that comparable noniridescent structural color has gone previously unnoticed in the case of zebrafish (63) and lizards (25).

Although the range of structural colors described here is wide, it should be noted that in many species, these colors act in combination with pigmentary color. *Flabellina iodinea* is described as having “silvery reflectance” which enhances pigmentary color (6). However, silvery reflectance should only enhance pigmentary coloration if the silvery reflector lies below the pigment (such that light has multiple opportunities to be absorbed). Here, the photonic structure in Fig. 3 *A* and *B* was located at the surface of the animal and therefore lies above any pigments present in the body. Therefore, we suggest that guanine crystal stacks are responsible for the generation of specific hues in the species studied here. So, although these structural colors act synergistically with the pigmentary coloration, they not only enhance colors but also are responsible for generating them.

## Conclusions

Through optical and ultrastructural imaging, we have shown that nudibranchs from across the dorid and aeolid groups utilize guanine crystals for highly variable structural coloration. Based on imaging and simulations, we suggest that the difference in color of discrete “pixels” comes from a variation in guanine platelet size and stacking pitch. Different species utilize various length scales of guanine platelets to generate a range of colors. For example, *H. bullockii* exhibits structurally colored granules in the near-UV (ultra violet) range, due to a smaller pitch of guanine platelet stacks, while the white region of *B. stephanieae* displays granules across the visible range up to the red region. On top of this multilayer pitch modulation, we propose that *C. willani* and *C. annae* produce brilliant white coloration via the optical mixing of colored reflections from across the spectrum, utilizing an array of guanine multilayer stacks in combination with a scattering material. The misalignment of stacks in species such as *C. annae* allows for angle-independent coloration, which may be key for the aposematic function of color in these slow-moving animals, allowing them to deter predators from any viewing position.

Behavioral studies would be required to understand the aposematic role of guanine here, but it certainly imparts very bright colors onto the surface of these animals (up to 80% reflectance for *C. annae*) in a variety of striking colors. Subtle structural changes in multilayer guanine reflectors—capable of generating a broad spectrum of colors—play a key role in color diversity among nudibranchs in the two groups described here. Such architectures not only allow for bright colors to be achieved but also for multiple colors in the same species made from the same material, increasing the possibility of pattern diversity.

## Materials and Methods

### Phylogenetic Tree.

A phylogenetic tree was constructed using phyloT (https://phylot.biobyte.de), a web-based tool that generates trees based on the hierarchical taxonomy in the NCBI Taxonomy database. A list of the 14 relevant species and their associated NCBI Taxonomy IDs was used as the input. phyloT retrieved the taxonomic lineages for each entry and constructed the tree using these lineages and their hierarchical relationships.

NCBI taxonomic information integrates information from a variety of biological data sources, including genetic studies, morphological traits, databases, and published literature. Importantly, phyloT does not use sequence data or apply clustering algorithms. Taxa are instead grouped and ordered based on their positions within the taxonomy database.

The tree was exported in Newick format for visualization using Interactive Tree of Life (https://itol.embl.de) and subsequently reproduced using Adobe Illustrator.

### Photography and Digital Microscopy.

A Keyence VHX-7100 digital microscope was used to take high magnification images of live sea slugs (*SI Appendix*, Fig. S11). A Nikon D7500 Single-Lens Reflex camera was used to take macroscopic images of the sea slugs.

### Optical Imaging and Spectroscopy.

Microspectroscopy was carried out using a customized Zeiss Axio Scope A1 microscope, fitted with a Pixelink PL-D725CU-T high frame rate camera, which was color calibrated with a white diffuser (Labsphere USRS-99-010). A Zeiss HAL100 halogen lamp was used as a light source. For acquiring spectra, the microscope was coupled to an Avantes AvaSpecHS2048 spectrometer using an Avantes FC-UVIR50 (50 µm core diameter) multimode optical fiber. Spectra were taken using water immersion objective lens 40× [Zeiss, WN-Achroplan 40× (NA 0.75, FWD 2.1 mm)]. Spectra were referenced to a protected silver mirror (PF10-03-P01, with >97.5% reflectance for 450 nm to 2 µm).

The samples were placed on a glass slide, and a coverslip was placed on top. Samples were then imaged on a Keyence VHX-7100 digital microscope before being transferred for spectra. Granule diameters were measured as the mean of 35 discrete granules (optically distinct islands of a single color) from four nonoverlapping regions of the body, from 1 to 2 individuals per species. Seawater was used as the medium between the water immersion objective and coverslip to ensure index matching. Spectra were taken in the Köhler illumination configuration, with the aperture diaphragm close to fully closed to reduce the collection of stray light from undesired areas. An image of the fiber collection area was taken prior to spectra using an external lamp, and a circle representing this area overlayed on the PixelLink imaging software to accurately know from which area spectra were being taken (*SI Appendix*, Fig. S12).

### Raman Spectroscopy.

Raman spectroscopy was performed on cryo-cut, unfixed, unstained room temperature 14-µm-thick sections of sea slug tissue. Samples were cut at −20 °C using a Leica CM3050 S cryostat. An alpha300 R—Raman Imaging Microscope (Oxford Instruments) equipped with a Ultra-High Throughput spectrometer was used to take Raman spectra. Spectra were taken with a 100× objective (Nikon) in confocal mode. A near-IR laser (*λ* = 785 nm) was used for all species due to fluorescence induced by the green laser in aeolid samples. Green laser (532 nm) Raman spectra are shown in *SI Appendix*, Fig. S13. For each species, the desired region of interest was located on the sections using a Keyence microscope in full ring mode (*SI Appendix*). The sample was then transferred to the Raman microscope and the desired region of interest was measured in at least three positions with an integration time of 60 s for each. These positions were averaged and processed using the Project *FIVE*+ software (Oxford Instruments).

### FIB SEM.

These data were taken on a Thermo Fisher Hydra Bio Plasma-FIB. 3D reconstruction was done using both the Thermo Scientific Amira Software and Object Research Systems (ORS) Dragonfly software. The sample is a high-pressure frozen region of blue dorsal tissue of *C. annae*. The desired areas of tissue were cut from the body of a sedated slug with a scalpel and under a stereo microscope (Zeiss). Immediately after dissection, the sample was placed into a B gold-coated copper freezer hat (BALTIC preparation, Wetter, Germany) and 10 wt% dextran (Sigma, 31390) was added as a cryoprotectant. A second freezer hat was added in a mirror configuration to enclose the sample, giving a total cavity thickness of 0.6 mm. Within approximately 5 min of dissection, the sample in this sandwich combination was cryoimmobilized using a high-pressure freezer (HPM100, Leica Microsystems).

### FIB SEM Data Acquisition.

Sequential plasma focused ion beam milling and imaging (also often referred to as “Slice & View”) were conducted using the Helios 5 Hydra DualBeam system (Thermo Fisher Scientific, Inc., Waltham, MA). Secondary electron images were acquired with the Thru-lens detector in immersion (ultrahigh resolution) mode. Samples mounted on a 35° pretilt shuttle were loaded onto the microscope cryostage. Experiments utilized Argon ions at 30 kV. A protective, nonconductive platinum-organic coating was deposited on the sample surface using a Gas Injection System (GIS), sandwiched between two sputter-coated conductive platinum (Pt) layers before milling trenches. Sputter coating was performed at 12 keV and 250 nA, with layer thicknesses of approximately 5 to 10 nm, while the GIS coating was around 1 µm thick.

Upon identifying an area of interest, a trench was milled using 30 kV, ~7 nA, followed by further polishing with 2 nA and 0.74 nA currents. This process enabled rapid verification of the area of interest within the milled region and facilitated quality assessment. Subsequently, a trench was milled on the left side of the initial front trench to mitigate charging effects, which typically manifest as “dark flares” from the left side of the image. A portion of the dark region near the left trench was intentionally included in the imaging area to serve this purpose.

Volume data acquisition was performed using Thermo Scientific™ Auto Slice and View 5 software under the following conditions: Milling parameter—30 kV, 200 pA; slice thickness—20 nm; SEM landing energy—1.2 keV; beam current—13 pA; dwell time—50 ns; line integration—150; lateral pixel size—5 nm × 5 nm.

### FIB SEM Data Processing.

The data were imported into Thermo Scientific™ Amira software for postprocessing and aligned using a rigid translational approach. Denoising was initially performed using a simple Gaussian filter with settings of 1,1 and a kernel size of 2. To further enhance the separation of crystals from the surrounding tissue, a Non-Local Means denoising filter was applied with the following settings: Search Window size = 30 px, Local Neighborhood = 2 px, and Similarity Value = 0.4. The Match Contrast module was utilized to normalize contrast brightness across the entire stack.

Segmentation of the crystals was conducted in Amira’s classic segmentation room and ORS Dragonfly. In Amira, this was done using a combination of the Threshold module and manual refinement of the resulting selection. For instances where multiple color volumes were displayed, a labeling step was performed to assign different colors to objects without common pixels. The Label Analysis module was used to extract volume and surface measurements of the crystals. In Dragonfly segmentation was conducted using a custom uNet model, this model was trained using three SEM images with thresholding and subsequently manually corrected.

Finally, Volume Rendering was applied to the segmented data to visualize it in 3D, and measurements were performed in 3D using Amira’s 3D measuring tool. Both stacks, containing 13 and 295 slices, respectively, were processed using the same protocol described above.

### Freeze Substitution.

The freshly cut tissue (cerata of *B. stephanieae*, pink foot of *H. bullockii*) of nudibranchs was sandwiched individually between two type B gold-coated copper high-pressure freezer carriers (BALTIC preparation, Wetter, Germany) with the addition of sea water. The carriers were positioned in a mirror combination to allow a total cavity thickness of 0.6 mm. The sandwiched samples were cryo-immobilized in an Electron Microscopy Instrumentation for Cryo-Embedding high-pressure freezing machine (Leica Microsystems, Vienna, Austria) within 10 min after dissection. Freeze substitution was carried out in an automatic freeze substitution unit (Leica Microsystems, AFS). The samples were first transferred under liquid nitrogen to tubes containing a freeze substitution medium composed of 2 wt% OsO_4_, 0.5 wt% glutaraldehyde, and 1.5 wt% water in acetone. Tubes were placed in AFS which was set at −120 °C. They were subsequently warmed to −85 °C at a rate of 17.5 °C/h and held at −85 °C for 103 h, then warmed to −20 °C at a rate of 7.2 °C/h, followed by a hold at −20 °C for 12 h, and a final heating to 4 °C at a rate of 2.7 °C/h followed by holding at 4 °C for 24 h.

The samples were rinsed with acetone, infiltrated with Embed 812 epoxy resin for 3 d. The resin polymerization was done for 24 h at a curing temperature of 65 °C.

### FDTD Simulations.

FDTD simulations were performed with a commercial solver (Ansys Lumerical). The 3D cryo-FIB-SEM data were imported as an isotropic material and embedded in a 2D FDTD simulation with PML boundary conditions in all directions, a mesh size of 15 nm, and a total simulation time of 2 ps. The imported structure was illuminated with a broadband diffracting plane wave and the power was monitored with a linear DFT monitor placed behind the source in the incident direction to collect reflected light (*SI Appendix*, Fig. S14). For simulating the reflectance of individual guanine domains, source and detector were positioned as close as possible to the structure to simulate high-NA collection and the simulation window was 2 µm × 2 µm. To calculate the angular dependence of the whole structure, the source and detector were placed approximately 5 µm away from the center of the structure, which is rotated by a total of 360° in steps of 10°, in a simulation area of 11 µm × 11 µm. At each angle of rotation, the 2D simulation was run over all z-planes of the image dataset and the resulting spectra were averaged and smoothed with a savitzky–golay filter. The maximum reflectance of each resulting spectrum was retrieved, and the center wavelength was calculated by fitting a Gaussian to the averaged and smoothed reflectance spectrum. Refractive indices were n = 1.83 for the guanine layers and n = 1.34 for the surrounding tissue.

### Transfer Matrix Simulations.

The reflectance spectra used to generate *SI Appendix*, Fig. S8 were obtained via numerical simulations using a modified StackModel routine from the open-source PyLlama Python library (61). In *SI Appendix*, Fig. S8*B*, simulations were performed by sweeping the incidence angle from 0° to 45° for a multilayer system consisting of N = 10 stacks, each composed of platelets with a thickness of 55 nm and a spacing of 70 nm. In *SI Appendix*, Fig. S8*C*, reflectance was simulated across the visible–UV spectral range, using a leptokurtic distribution of incidence angles with the center of mass around normal incidence. The model assumed an isotropic multilayer system, also consisting of N = 10 stacks, where each platelet had a thickness varying between 50 nm and 90 nm, separated by gaps ranging from 50 nm to 110 nm. To better mimic experimental conditions, Gaussian white noise was added to the platelet thickness, with a threshold of less than 10%. In all cases, the system followed a sandwich heterostructure composed of two materials with refractive indices n_A_ = 1.83 and n_B_ = 1.34 (background), respectively.

### Handling and Ethics.

1-2 individuals of each species were studied. All species were bought from online aquarium shops (Rifix, Germany; Coral & Fish Store and De Jong Marinelife, Netherlands) apart from *S. neapolitana* which was collected in an intertidal zone in the Eastern Atlantic. Dorid nudibranchs originate from the western Indo-Pacific, and this study has been acknowledged by the Republic of the Philippines Department of Biodiversity and Natural Resources, Biodiversity Management Bureau. Animals were studied immediately after arrival. Tropical species were kept at 26 °C and temperate species were kept at 18 °C in separate aquaria. All specimens were treated with best practices; this includes sedating of animals prior to experiments and regular replacement and aeration of seawater. None of the listed species are protected under Convention on International Trade in Endangered Species of Wild Fauna and Flora, International Union for Conservation of Nature, or national endangered species acts.

## Supplementary Material

Appendix 01 (PDF)

Movie S1. Digital microscope (Keyence VHX-7100) video (50× magnification) of a *Chromodoris annae* individual displaying bright blue, angle independent structural coloration, along with white, black and yellow coloration.

## Data Availability

Images, Raman spectra, optical spectra, simulations, EM images data have been deposited in Edmond (https://doi.org/10.17617/3.CDDKLB) (64).
